# Biocontrol Mechanisms of a Chinese *Heterorhabditis indica* Strain Against *Tuta absoluta*: Virulence Assay and Time-Course Transcriptomics of Host Immune Responses

**DOI:** 10.3390/insects17030240

**Published:** 2026-02-26

**Authors:** Shuocheng Zeng, Hang Yu, Raquel Campos-Herrera, Xingru Chen, Wencai Lu, Xingyue Li

**Affiliations:** 1 Institute of Plant Protection, Sichuan Academy of Agricultural Sciences, Chengdu 610066, China; zengshuocheng@scsaas.cn; 2 Chongqing Academy of Agricultural Sciences, Chongqing 401329, China; chenxingru@cqaas.cn; 3 Horticultural Crops Germplasm Innovation and Utilization Key Laboratory of Sichuan Province, Horticulture Research Institute, Sichuan Academy of Agricultural Sciences, Chengdu 610066, China; yuhang5810@163.com; 4 Instituto de Ciencias de la Vid y del Vino (CSIC, Universidad de La Rioja, Gobierno de La Rioja), Finca La Grajera Ctra. Burgos Km. 6 Salida 13 Lo-20, 26007 Logroño, Spain; raquel.campos@icvv.es

**Keywords:** *Tuta absoluta*, *Heterorhabditis indica*, biological control, transcriptomics, immunity, parasitism

## Abstract

The South American tomato pinworm, *Tuta absoluta*, is a devastating invasive pest that threatens global tomato production, while entomopathogenic nematodes (EPNs) represent promising biocontrol agents. Because a detailed understanding of the molecular basis of the insect immune response is crucial for uncovering how hosts detect and counteract nematode infection, such knowledge may reveal weaknesses exploitable for improved control strategies. However, the molecular mechanisms governing the immune interaction between this pest and EPNs remain poorly understood This study investigated the biocontrol potential of a native Chinese EPN strain, *Heterorhabditis indica* CQ7-2, against *T. absoluta* and delineated the host’s molecular immune responses via a time-course transcriptomic analysis. Bioassays revealed that *H. indica* CQ7-2 LC_50_ was 1.35 IJs per larva. Comparative transcriptome profiling of larvae revealed that the EPN infection was associated with transcriptional patterns consistent with immunosuppression. Key genes involved in humoral and cellular immunity were significantly suppressed during the early and middle infection stages. Although a widespread upregulation of immune genes occurred after 18 h post-infection (hpi), it was insufficient to prevent host mortality. These findings demonstrate that the virulence of *H. indica* CQ7-2 is underpinned by associated with modulation of key immune pathways, leading to an ineffective defense response. This work provides deep insights into the molecular arms race between an invasive pest and a native EPN, supporting CQ7-2 as a promising biocontrol agent and providing a framework for understanding host-EPN interactions.

## Simple Summary

The invasive South American tomato pinworm, *Tuta absoluta*, is a major threat to tomato crops worldwide. As conventional control methods face challenges, there is a growing need for sustainable alternatives. We isolated a native strain of entomopathogenic nematode, *Heterorhabditis indica* CQ7-2, from local soils and evaluated its potential. Laboratory bioassays demonstrated that this strain is effective at infecting and killing the pest larvae, with a median lethal concentration of only 1.35 infective juvenile nematodes per larva. Further time-course transcriptomic analysis revealed that the nematode impairs the insect’s immune system, preventing it from mounting a successful defense. This work demonstrates the pathogenicity of *H. indica* CQ7-2 and its association with host immune suppression, supporting its potential as a biocontrol agent for further development.

## 1. Introduction

The South American tomato pinworm, *Tuta absoluta* (Meyrick) (Lepidoptera: Gelechiidae), represents one of the most rapid and extensive invasions by an agricultural pest in the 21st century. Originally from Peru, *T. absoluta* spread throughout South America by the 1960s, reached Spain in 2006, and subsequently colonized over 110 countries across Europe, Africa, and Asia within a decade [1,2]. This alarming expansion has been fueled by several factors, including the international trade of infested seedlings, wind-assisted dispersal of adults (up to 100 km/year), and remarkable adaptation to diverse climates from Mediterranean greenhouses to subtropical open fields [3,4]. The larval endophytic mining behavior of *T. absoluta* results in yield losses of 80–100% in unprotected tomato crops, primarily by reducing photosynthetic capacity by 30–70% and facilitating secondary infections from pathogens such as *Botrytis cinerea* (Helotiales: Sclerotiniaceae) [5,6]. *T. absoluta* has caused significant economic losses globally (e.g., over $5 billion annually), with severe impacts in China since its invasion in 2017 [7,8,9].

The exceptional invasiveness of *T. absoluta* stems from a suite of complementary traits: its high reproductive potential (10–12 generations per year), broad host range (including eggplant, potato, and various wild Solanaceae), and its cryptic larval behavior. The larvae develop entirely within leaf mesophyll or fruit tissues, effectively evading both contact insecticides and the detection by natural enemies [5]. Compounding this challenge, the pest has evolved extraordinary adaptability to chemical controls, with documented resistance to multiple insecticide classes in 15 countries. This resistance arises from enhanced detoxification mechanisms (e.g., monooxygenases, esterases) and target-site mutations (e.g., acetylcholinesterase, ryanodine receptors) [10,11,12,13], with field populations in Kenya and Iran showing 35–162-fold resistance to pyrethroids, rendering conventional sprays ineffective [14,15]. Even microbial biopesticides like *Bacillus thuringiensis*, while still effective in some regions, are already facing emerging resistance—for instance, populations in Gansu Province, China, exhibit nearly 5-fold higher resistance levels compared to those in Yunnan Province [16,17]. This widespread, multi-layered resistance crisis not only undermines existing management strategies but also highlights an urgent need for non-chemical alternatives that bypass evolved detoxification pathways—and entomopathogenic nematodes (EPNs) emerge as a particularly promising candidate, having been proven effective against *T. absoluta* in Moroccan field trials [18].

Efforts at augmentative biological control combining the parasitoid *Trichogramma brassicae* (30 parasitoids weekly) and predator *Nesidiocoris tenuis* (released before *T. absoluta*) increased the undamaged fruit rate of tomato crops from 23.91% to 87.41% in plastic greenhouses [19]. However, similar approaches are generally limited by several factors: (1) poor establishment in open-field systems due to climatic sensitivity (e.g., *N. tutae* activity declines below 15 °C), (2) intraguild predation (e.g., *Macrolophus pygmaeus* preying on *Trichogramma* pupae), and (3) inconsistent host location efficiency in dense canopies [20,21]. In contrast, EPNs from the genera *Steinernema* and *Heterorhabditis* offer distinct advantages in managing *T. absoluta*. Their infective juveniles (IJs) actively seek hosts using CO_2_ gradients and herbivore-induced plant volatiles, allowing them to penetrate leaf mines and soil-dwelling pupal chambers [22,23]. They kill their hosts via symbiotic bacteria (*Xenorhabdus* and *Photorhabdus*), which evade insect immune responses through cytotoxic compounds (e.g., stilbenes, indoles), causing septicemia within 48 h [24,25,26]. Crucially, EPNs exhibit minimal non-target effects on pollinators and vertebrates, align with organic certification standards, and display no cross-resistance with chemical insecticides [27,28,29,30]. Greenhouse condition trials in Spain have demonstrated 87–95% reduction of insect infection using *Steinernema feltiae*, significantly outperforming standard chemical control rotations [31].

Despite the promising potential of EPNs for *T. absoluta* management, three interconnected barriers hinder their optimal deployment. First, while most commercially available EPNs (e.g., *Steinernema carpocapsae*, *S. feltiae*, *Heterorhabditis bacteriophora*) are widely used for controlling lepidopteran pests, no products specifically optimized for *T. absoluta*. For instance, certain local isolates of EPN from Spain demonstrated over 85% larval mortality against *T. absoluta* in laboratory assays, performing comparably to the commercially available EPNs from Koppert; yet, systematic screening of Asian-native EPNs for *T. absoluta* control remains limited [32]. Second, EPNs are particularly vulnerable to environmental stressors, including temperature, humidity, and UV radiation [33,34]. Lastly, the molecular mechanisms underlying *T. absoluta*’s immune responses to EPN infection is critically relevant for advancing EPN-based biocontrol strategies—especially given the pest’s global invasiveness, widespread insecticide resistance, and the urgent need for sustainable alternatives. While studies on other lepidopterans indicate that nematode penetration triggers phenoloxidase activation, antimicrobial peptide production (e.g., gloverin), and hemocyte encapsulation, the specific defense repertoire of *T. absoluta* and how EPNs may counter these defenses remain poorly characterized [35,36,37].

To address these critical gaps, our study prioritized screening indigenous Chinese EPNs with enhanced virulence against *T. absoluta* larvae, focusing on the locally adapted *Heterorhabditis indica* strain CQ7-2. This strain exhibits remarkable climatic resilience, maintaining over 88% survival after a 40-h exposure to 36 °C, significantly surpassing commercial counterparts. It also demonstrates a high reproductive yield of 4.50 × 10^5^ infective juveniles per *Galleria mellonella* host and exhibits potent virulence [38]. Through meticulously controlled laboratory assays, we quantified the dose-mortality relationships for CQ7-2, establishing baseline virulence parameters. Furthermore, we conducted the first transcriptomic analysis of *T. absoluta* immune responses specifically to *H. indica* infection, profiling key defense pathways following CQ7-2 challenge. This integrative approach bridges molecular insights with field-applicable biocontrol strategies.

## 2. Materials and Methods

### 2.1. Insect Rearing and EPN Maintenance

A laboratory colony of *T. absoluta* was established from individuals collected in tomato greenhouses in Xindu District, Chengdu City, Sichuan Province, China. The colony had been maintained under controlled conditions for over five consecutive generations prior to experimentation. Tomato plants (*Solanum lycopersicum*), dwarf variety, were cultivated in pots within a climate-controlled chamber and served as the host plants for *T. absoluta*. Adult moths and potted tomato plants were housed together in rearing cages (40 cm × 40 cm × 40 cm) constructed from 120-mesh nylon netting. The insects were transferred to fresh host plants when the existing foliage was extensively mined or showed signs of deterioration. Both the plant growth chamber and the insect rearing cages were maintained at 26 ± 1 °C, 60% relative humidity, and a photoperiod of 14: 10 h (light: dark). To facilitate colony expansion, newly emerged adult moths were introduced into cages containing fresh potted tomato plants and provided with cotton wicks soaked in 10% (*v*/*v*) honey water as a supplementary nutrient source.

The EPN *H. indica* strain CQ7-2, originally isolated from soil in Dazu District, Chongqing, China, was used in this study. The nematode colony was maintained in the laboratory using last-instar larvae of the greater wax moth, *G*. *mellonella*, as a propagation host according to standard procedures. Infective juveniles (IJs) were harvested from deceased hosts using White traps. The newly emerged IJs were collected, surface-cleaned, and subsequently stored in aqueous suspension in tissue culture flasks at 14 °C in darkness for long-term preservation, with those intended for experiments used within two weeks to maintain viability.

### 2.2. Bioassay

To evaluate the biocontrol potential of the indigenous EPN *H. indica* strain CQ7-2 against *T. absoluta*, a concentration-mortality bioassay was conducted under controlled laboratory conditions. The pathogenicity of the nematode was assessed by exposing the last-instar larvae of *T. absoluta* to a gradient of IJ concentrations: 5, 10, 20, 40, and 80 IJs per larva, with a control group where larvae were exposed to sterile water instead of IJ suspensions.

Aqueous suspensions of IJs were prepared at corresponding concentrations of 250, 500, 1000, 2000, and 4000 IJs/mL. For the assay, 20 µL of each suspension was dispensed onto a filter paper disc placed at the bottom of each well in a 24-well culture plate. One last-instar *T. absoluta* larva was randomly assigned into each well. Larval survival was monitored and recorded at 12-h intervals post-infection, with final survival rates calculated upon completion of the bioassay to minimize potential observer bias. The cause of death was confirmed as EPN infection by the subsequent emergence of IJs from all observed cadavers.

The experimental design included three independent biological replicates per IJ concentration, with each replicate utilizing IJ suspensions derived from distinct culture batches to account for biological variability. Each biological replicate comprised three technical replicates (i.e., 15 larvae per concentration), which were processed simultaneously using the same nematode batch under identical conditions. The mortality data from technical replicates within each biological replicate were first checked for consistency using the Log-rank test (*p* > 0.05 for homogeneity). Inter-replicate consistency of biological replicates was confirmed by Log-rank tests (all *p* > 0.05) (Table S1). Subsequently, the validated data were pooled, resulting in a total of 45 larvae assessed per IJ concentration for robust statistical analysis.

The concentration-mortality response was analyzed using probit analysis in SPSS software (version 21.0) to determine the median lethal concentration (LC_50_) and the median lethal time (LT_50_) of strain CQ7-2 against *T. absoluta* larvae. Data visualization and secondary graphical analysis were performed using GraphPad Prism software (version 10).

### 2.3. RNA Isolation

Total RNA was extracted from *T. absoluta* larvae at three critical post-infection time points (6, 12, and 18 h) following exposure to 80 IJs of *H. indica* strain CQ7-2—a dose selected based on our bioassay results, as it induced host mortality within a narrow time window (22–26 h post-infection), thereby ensuring strong correlation among biological replicates across the selected sampling time points. Uninfected larvae served as controls. For each time point, three independent biological replicates were established. Larvae for each replicate were first surface-sterilized, then ten randomly selected last-instar individuals were pooled and placed into a single 2 mL reinforced tubes containing five 3-mm steel beads. The tubes were immediately flash-frozen in liquid nitrogen, and stored at −80 °C until all samples across all experimental groups were collected.

Frozen samples were homogenized using a frozen tissue homogenizer. Due to the high lipid content characteristic of lepidopteran larvae, which can pose challenges for nucleic acid purification, total RNA was isolated using the GOONIE Cell/Tissue RNA Extraction Kit (GOONIEBIO, Guangzhou, China, Cat# 400-105), strictly following the manufacturer’s instructions. This kit is designed to effectively handle tissues rich in fats and other secondary metabolites. The final RNA pellet was resuspended in 35 μL of sterile, nuclease-free water.

To eliminate potential genomic DNA contamination, the extracted RNA was treated with DNase I (New England Biolabs, Ipswich, MA, USA, Cat# M0303L). The purity of the RNA was assessed by measuring the A260/A280 ratio using a Nanodrop™ OneC spectrophotometer (Thermo Scientific, Waltham, MA, USA). Samples with ratios between 1.8 and 2.1 were considered acceptable. RNA integrity was validated using the LabChip GX Touch system (Revvity, Waltham, MA, USA), which provides an RNA Integrity Number (RIN) equivalent score. Finally, the concentration of the qualified RNA samples was accurately quantified using a Qubit 3.0 fluorometer with the Qubit™ RNA Broad Range Assay kit (Thermo Scientific, Waltham, MA, USA, Cat# Q10210), ensuring precise measurement for subsequent library construction.

### 2.4. Library Preparation and RNA Sequencing

Strand-specific sequencing libraries were constructed using the KC-Digital™ Stranded mRNA Library Prep Kit (Seqhealth Tech. Co., Ltd., Wuhan, China), following the manufacturer’s instructions. Briefly, mRNA was enriched from total RNA using oligo(dT) magnetic beads and subsequently fragmented. First-strand cDNA synthesis was performed using random hexamer primers and reverse transcriptase, followed by second-strand synthesis. The resulting double-stranded cDNA underwent end repair, adenylation, and ligation with indexed adapters. The adapter-ligated fragments were then amplified by PCR to generate the final sequencing libraries.

Qualified libraries with distinct indices were pooled in equimolar amounts. Pooled libraries were subjected to paired-end sequencing (PE150) on the DNBSEQ-T7 platform (MGI) at Seqhealth Tech. Co., Ltd. This process generated an average of approximately 50 million raw reads per sample, providing sufficient depth for subsequent transcriptomic analysis.

### 2.5. Sequencing Data Analysis

The analysis of raw sequencing data commenced with quality control and adapter trimming using fastp (v0.23.2) to ensure the reliability of downstream analyses. Subsequent processing leveraged Unique Molecular Identifiers (UMIs) for precise quantification. Specifically, umikit (v1.0) was used to identify and extract UMI sequences from clean reads using anchor sequences. Reads possessing valid 5′ and 3′ UMI anchors were classified as valid UMI reads, among which those with insert lengths shorter than 15 nucleotides were discarded.

The remaining high-quality UMI reads were aligned to the *T. absoluta* reference genome (GenBank assembly accession: GCA_027580185.1) using STAR software (v2.5.3a) with default parameters. To mitigate PCR duplication biases and enhance accuracy, UMI-based deduplication and error correction were performed using a customized gencore (v1.0) pipeline. This process involved clustering reads that shared identical genomic alignment coordinates and UMI sequences.

Read counts for genes and transcripts were quantified separately using featureCounts (from the Subread package v1.5.1), targeting exon regions for gene-level analysis and individual transcripts for isoform-level quantification. The resulting count matrix was used to identify differentially expressed genes (DEGs) and transcripts between experimental groups with the edgeR package (v3.40.2), applying a significance threshold of FDR < 0.05, *p*-value ≤ 0.05 and log_2_ fold change (|log_2_FC|) ≥ 1. To elucidate the biological functions of the identified DEGs, Gene Ontology (GO) and Kyoto Encyclopedia of Genes and Genomes (KEGG) pathway enrichment analyses were conducted using KOBAS software (v2.1.1), with a statistical significance cutoff of *p*-value ≤ 0.05. Finally, to capture dynamic transcriptional changes, time-series gene expression patterns were classified using unsupervised clustering with the R package Mfuzz (v2.58.0).

### 2.6. RT-qPCR Validation

To assess the reliability of the transcriptome sequencing results, the expression levels of eight candidate genes, which exhibited significant differential expression across the time-course infection experiment, were validated using quantitative real-time PCR (RT-qPCR) with three biological replicates per treatment group (Figure S1).

First-strand cDNA was synthesized from 1 μg of total RNA per sample using a commercial MightyScript Plus First Strand cDNA Synthesis Master Mix (Sangon Biotech, Shanghai, China, Cat# B639252), following the manufacturer’s protocol. The resulting cDNA was then diluted ten-fold with nuclease-free water and used as the template for subsequent qPCR reactions.

qPCR was performed using the SGExcel Universal SYBR qPCR Mix (Sangon Biotech, Shanghai, China, Cat# B532955) on a qTower^3^ real-time PCR system (Analytik Jena, Jena, Thuringia, Germany). All primer pairs were designed using the Oligo 7.0 software. The specificity of each primer pair and the amplification efficiency were confirmed through both the analysis of a melting curve and a standard curve generated from a serial dilution of pooled cDNA samples. The total reaction volume for qPCR was 20 μL. The thermal cycling conditions were set as follows: an initial denaturation at 95 °C for 3 min, followed by 40 cycles of denaturation at 95 °C for 10 s, and a combined annealing/extension step at 60 °C for 30 s. For each of the four time points, three biological replicates (established from separate batches of EPN cultures) were analyzed, and each reaction was run in triplicate (technical replicates) for every primer set.

The relative expression levels of the target genes were normalized to the endogenous control gene, *elongation factor-1 alpha* (*EF1α)*, and calculated using the comparative 2^(−ΔΔCt)^ method [39]. The sequences of all primers used in this study are provided in Table S2.

### 2.7. Data Access

The raw sequence data that were generated in the course of this research are made publicly available. Paired-end sequencing data of the *T. absoluta* transcriptomes have been deposited in the Genome Sequence Archive (GSA) at the CNCB-NGDC, under BioProject accession number PRJCA054879 (GSA: CRA036293), which is publicly accessible at https://ngdc.cncb.ac.cn/gsa (accessed on 2 January 2026) [40,41].

## 3. Results

### 3.1. Virulence of H. indica Strain CQ7-2 Against T. absoluta

**Figure 1:**
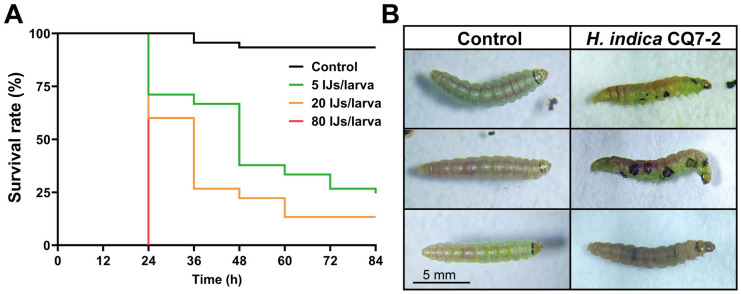
Pathogenicity of *H. indica* strain CQ7-2 against *T. absoluta*. (**A**) Survival rates of last-instar *T. absoluta* larvae infected by IJs of *H. indica* strain CQ7-2 (*n* = 45). (**B**) Phenotype at 24 hpi of last-instar *T. absoluta* larvae, showing locally or globally activated melanization responses.

The survival curve, constructed based on mortality rates, demonstrated that *H. indica* strain CQ7-2 exhibited high virulence against *T. absoluta* larvae (Figure 1A). At the highest concentration of 80 IJs/larva, 100% mortality was achieved within 24 h post-infection. A concentration of 20 IJs/larva resulted in a maximum mortality of 86.7% by 60 h, while even the lowest concentration of 5 IJs/larva caused a mortality rate of 75.6%. The median lethal concentration (LC_50_) of *H. indica* strain CQ7-2 against the last-instar *T. absoluta* larvae was calculated to be as low as 1.35 IJs/larva (Table S3). The reliability of this extrapolated estimate is supported by two factors: the substantial mortality (75.6%) caused by the lowest tested dose, and a well-fitting probit model. The model’s adequacy is confirmed by a non-significant goodness-of-fit test (χ^2^ = 2.196, df = 3, *p* = 0.533), with a dose-response slope of 1.113 (±0.303 SE), suggesting its appropriateness for the extrapolation.

Shortly after death, the larval cadavers became flaccid, and a localized cuticular melanization (darkening) response was frequently observed (Figure 1B). Subsequently, the color of the cadavers transitioned from yellowish-brown or tan to dark red, a pattern consistent with proliferation of the symbiotic bacteria (known to colonize EPN-infected hosts) [42].

### 3.2. Overview of RNA-Seq and Differentially Expressed Genes

**Figure 2:**
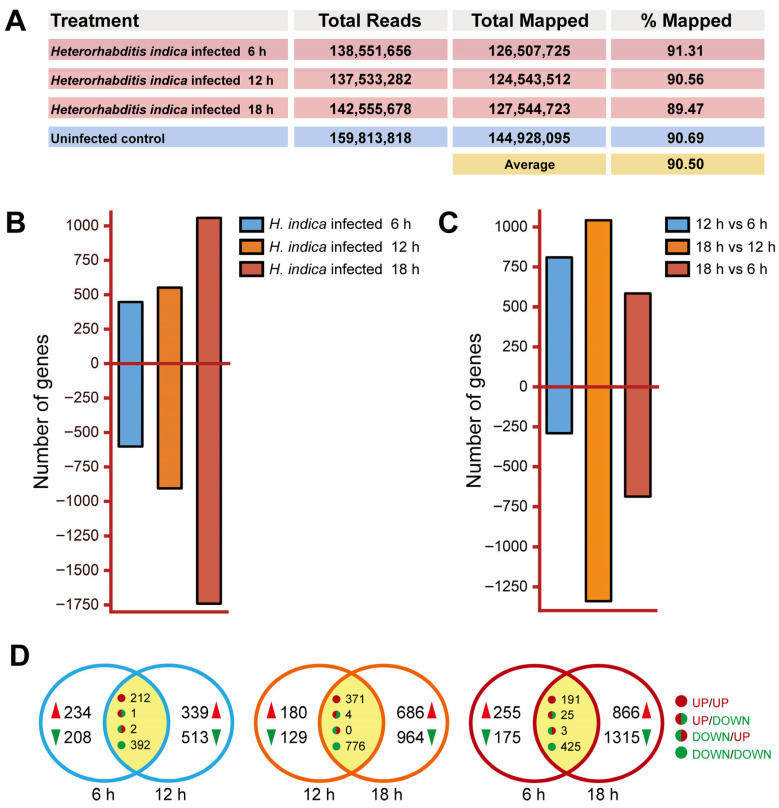
Dynamic transcriptomic profiling of *T. absoluta* larvae in response to *H. indica* CQ7-2 infection. (**A**) Summary of RNA-seq data, showing the total number of sequencing reads and the percentage mapped to the *T. absoluta* genome for each sample. (**B**) Number of DEGs that were up-regulated or down-regulated at each infection time point compared to the uninfected control. (**C**) Number of DEGs identified from pairwise comparisons between the different infection time points (6, 12 and 18 h post-infection). (**D**) Venn diagrams showing the overlap of DEGs between infection time points. Up-regulated and down-regulated genes are denoted in red and green, respectively. The expression patterns for overlapping genes are defined as follows: UP/UP (upregulated at both time points), UP/DOWN (upregulated at the earlier time point but downregulated at the later one), DOWN/UP (downregulated at the earlier time point but upregulated at the later one), and DOWN/DOWN (downregulated at both time points).

High-quality transcriptomic data were obtained, with biological replicates clustering tightly together and distinct separation among the four treatment groups clearly observed along the temporal gradient (Figures S2 and S3). Sequencing reads showed high alignment efficiency, mapping to 90.50% of the *T. absoluta* genome (Figure 2A). The number of differentially expressed genes (DEGs) increased progressively with infection time. The highest number was observed at 18 hpi. Specifically, relative to controls, 1048 DEGs were identified at 6 hpi, which increased 1.39-fold to 1448 DEGs by 12 hpi, and further surged 1.92-fold to 2799 DEGs by 18 hpi (Figure 2B). Pairwise comparisons between infection time points revealed that the greatest transcriptional shift occurred between 12 and 18 hpi, as evidenced by the markedly higher number of DEGs for this contrast compared to the 6 h vs. 12 h or 6 h vs. 18 h comparisons (Figure 2C).

A comparative Venn diagram analysis detailed the dynamics of gene regulation across the infection timeline (Figure 2D). While a core set of genes showed consistent up- or down-regulation between adjacent time points, the majority of DEGs were uniquely regulated at specific stages. This temporal specificity became increasingly pronounced over time. Notably, the comparison between 12 and 18 hpi showed the largest expansion of uniquely regulated genes, with over a thousand genes being specifically up- or down-regulated only at 18 hpi. Conversely, the direct comparison between the earliest (6 hpi) and latest (18 hpi) stages showed minimal overlap, underscoring a profound and largely stage-specific transcriptional reprogramming throughout the infection. Collectively, these data demonstrate that the host’s transcriptional response is highly dynamic, culminating in a massive and distinct wave of gene expression changes between 12 and 18 hpi.

### 3.3. Gene Ontology Enrichment Analysis Identifies Critical Pathways Regulated by H. indica CQ7-2 in T. absoluta

**Figure 3:**
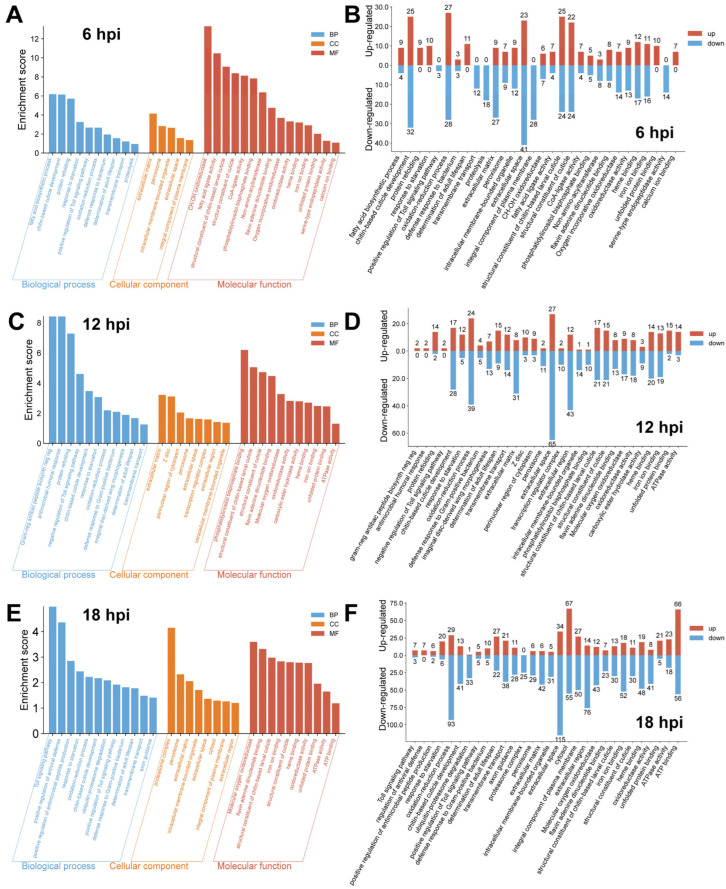
GO enrichment analysis of genes differentially expressed in *T. absoluta* larvae in response to *H. indica* CQ7-2 infection. The top enriched GO terms in the categories of biological process (BP, blue), cellular component (CC, orange), and molecular function (MF, red) are shown for the transcriptomes at 6 (**A**,**B**), 12 (**C**,**D**), and 18 (**E**,**F**) hours post-infection (hpi), with red (up-regulated) and blue (down-regulated) columns per time point respectively displaying the enrichment scores and the numbers of up- and down-regulated genes.

To identify the major biological processes, cellular components, and molecular functions affected by *H. indica* strain CQ7-2 infection in *T. absoluta*, we performed gene ontology (GO) enrichment analysis at 6, 12, and 18 hpi (Figure 3). Our analysis revealed a progressive increase in the number of differentially expressed genes (DEGs) over time, with the most extensive transcriptional changes occurring at 18 hpi.

At early stages of infection (6 hpi), the host response was primarily characterized by alterations in genes related to cuticle development and fundamental metabolic processes, such as oxidation-reduction (Figure 3A,B). A notable suppression was observed in proteolysis, while cellular component terms highlighted changes in the extracellular space and matrix. Molecular functions enriched included structural constituents of the cuticle, indicating that structural integrity and basic metabolism were early targets.

By 12 hpi, the transcriptional response intensified, showing a marked shift towards the downregulation of metabolic processes like oxidation-reduction (Figure 3C,D). Defense-related processes emerged, and the upregulation of unfolded protein binding signified a growing cellular stress response. The continued enrichment of cuticle-related terms suggested persistent structural modulation.

At late infection (18 hpi), the number of regulated genes peaked, underscoring a widespread transcriptional reprogramming, with immune-related pathways becoming highly prominent (Figure 3E,F). Key enrichments included the Toll signaling pathway and defense response to Gram-positive bacterium, indicating a substantial activation of immune defenses. Concurrently, processes such as transmembrane transport and cuticle development remained significantly represented but were predominantly downregulated. In molecular functions, ATP binding was the most enriched category, reflecting considerable shifts in energy metabolism.

Temporal shifts in GO enrichment reflected the progression of infection: early (6 hpi) disruption of cuticle integrity and suppressed proteolysis; mid-stage (12 hpi) changes in transmembrane transport and emerging defense responses to Gram-positive bacteria; and late (18 hpi) widespread downregulation of oxidative stress pathways and upregulation of immune signaling (e.g., Toll pathway, enrichment score 4.98). Cuticle-related genes were consistently downregulated across all stages, suggesting sustained impairment of the host’s physical barrier.

These data showed that *H. indica* strain CQ7-2 infection causes significant and dynamic changes in the expression of protein-coding genes in *T. absoluta*, affecting critical biological processes such as oxidative stress, cuticle integrity, immune response, and metabolism. The progressive increase in gene numbers and the shift in regulation patterns over time suggest that the pathogen modulates host transcription to establish infection, with potential implications for host defense and survival.

### 3.4. KEGG Pathway Analysis Reveals Systemic Metabolic and Signaling Alterations Induced by H. indica CQ7-2 Infection

**Figure 4:**
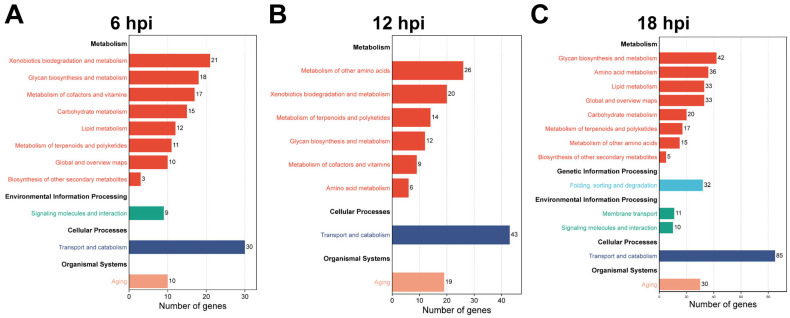
KEGG pathway analysis reveals systemic alterations induced by *H. indica* CQ7-2 infection over time. The bar charts display the number of DEGs mapped to the top enriched KEGG pathways at (**A**) 6, (**B**) 12, and (**C**) 18 hpi.

A predominant number of annotated DEGs were mapped to pathways categorized under Metabolism (indicated in red tones, Figure 4), underscoring a profound disruption of the host’s core biochemistry. This included significant gene representation in pathways for carbohydrate, amino acid, and lipid metabolism, consistent with the parasite’s demand for nutrients and the host’s compensatory or stress responses. Notably, pathways related to energy metabolism (e.g., oxidative phosphorylation) and xenobiotic biodegradation were also prominently featured, suggesting an intense cellular effort to cope with metabolic stress and potential toxins from the EPN’s symbiotic bacteria.

Beyond metabolism, a substantial proportion of DEGs were associated with pathways involved in Environmental Information Processing and Organismal Systems (Figure 4). Key among these were signaling pathways such as the MAPK, PI3K-Akt, and Toll and Imd signaling pathways, which are central to immune regulation and cellular stress responses. The enrichment of genes in these pathways aligns with the activation of immune defenses and the complex host-pathogen signaling interplay observed in the GO analysis. Furthermore, pathways related to transport and catabolism, as well as folding, sorting and degradation, were significantly represented, indicating extensive remodeling of cellular processes for immune effector production, tissue repair, and protein homeostasis.

The distribution pattern evolved over the course of infection, with the 18 hpi time point showing the most extensive gene involvement across the widest array of pathways (Figure 4C). This expansion reflects the escalating scale of physiological disruption as the infection progresses towards host mortality. Collectively, the KEGG pathway analysis delineates a comprehensive picture of infection-induced pathophysiology, where *H. indica* CQ7-2 infection co-opts host metabolism while simultaneously triggering defensive signaling cascades, ultimately leading to a global perturbation of the insect’s cellular systems.

### 3.5. Time-Series Clustering Delineates Opposing Regulation Strategies During the Host-Pathogen Interaction

**Figure 5:**
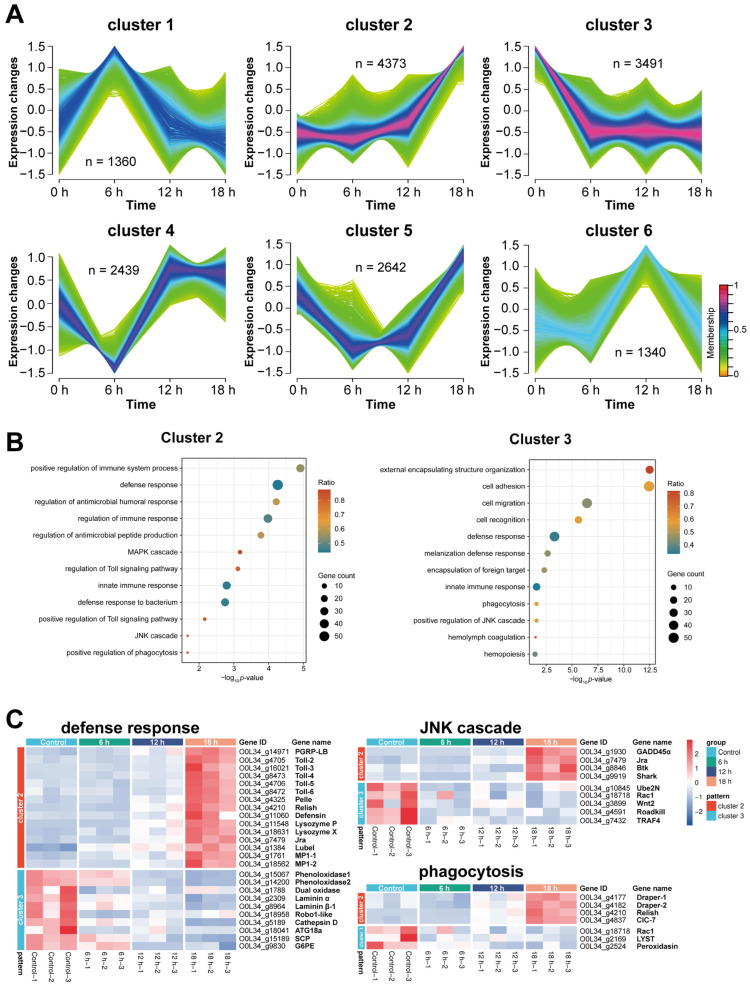
Time-series clustering and functional analysis of *T. absoluta* transcriptional responses to *H. indica* infection. (**A**) Expression trends of all genes, grouped into six distinct clusters by Mfuzz time-series analysis. The color of each line represents its membership value (the degree of similarity to the cluster center), as indicated by the color bar (range: 0–1). *n* represents the number of genes per cluster. (**B**) GO enrichment for Clusters 2 and 3. The top enriched biological processes for each cluster are displayed, with bubble size representing the number of genes and color indicating the enrichment significance. (**C**) Heatmaps depicting the expression patterns of genes annotated to the three key GO terms common to both clusters.

To decipher the dynamic progression of the host response, we performed time-series clustering on all differentially expressed genes across 0, 6, 12, and 18 hpi. Unsupervised clustering using Mfuzz partitioned the transcriptome into six distinct expression profiles (Cluster 1–6, Figure 5A). We focused subsequent analysis on Clusters 2 and 3, which contained the largest number of genes (4373 and 3491 genes, respectively), representing two dominant and opposing host transcriptional strategies. Cluster 2 genes exhibited a pattern of late, sharp upregulation, maintaining stable baseline expression through 12 hpi before a dramatic increase at 18 hpi. In stark contrast, Cluster 3 genes were characterized by rapid, early downregulation, with expression sharply dropping by 6 hpi and remaining suppressed thereafter (Figure 5A).

GO enrichment analysis of these two pivotal clusters revealed distinct functional landscapes (Figure 5B). In cluster 2, the analysis highlighted a pronounced enrichment for pathways centered on innate immune signaling and precise immunomodulation. Specifically, this cluster was dominated by terms such as “defense response”, “regulation of immune response”, and “positive regulation of immune system process”. The significant enrichment of signaling pathways like “regulation of Toll signaling pathway” and “positive regulation of Toll signaling pathway” further underscores its role in pathogen recognition and subsequent inflammatory cascade initiation. Additionally, the presence of “defense response to bacterium” and “regulation of antimicrobial peptide production” points to a specialized role in combating bacterial infections. Conversely, cluster 3 exhibited a functional profile geared towards cellular effector functions and structural remodeling. While it also showed enrichment in broad terms like “defense response”, its most striking enrichments were in processes such as “cell adhesion”, “cell migration”, and “external encapsulating structure organization”. This suggests a primary role in cell-cell interaction, tissue integrity, and wound healing. The enrichment in “hemolymph coagulation,” “encapsulation of foreign target,” and “melanization defense response” is particularly noteworthy, defining this cluster’s function in the physical encapsulation and neutralization of large pathogens, a hallmark of invertebrate immunity.

Notably, several immune and stress-related biological processes were significantly enriched in both clusters, creating a critical interface, including “defense response,” “regulation of JNK cascade,” and “phagocytosis.” This co-enrichment identified these functions as critical battlegrounds where the transcriptional regulation of key genes diverged radically—with cluster 2 potentially governing the sensory and signaling arm (e.g., Toll pathway, cytokine regulation), and cluster 3 executing the physical effector responses (e.g., adhesion, coagulation, encapsulation).

To visualize this molecular tug-of-war, we generated expression heatmaps for genes annotated to three key GO terms: defense response, JNK cascade, and phagocytosis (Figure 5C). Within each functional group, genes were partitioned based on their cluster affiliation, revealing a sophisticated division of labor in host immune strategies. In the defense response, the upper section (Cluster 2) includes key pathogen recognition and effector genes—such as peptidoglycan recognition protein (*PGRP-LB*), Toll pathway components (*Toll*, *Pelle*, *Relish*), the antimicrobial peptide *Defensin*, *lysozyme*, and *melanization enzyme* (*MP1-1*, *MP1-2*). These genes exhibited initial suppression followed by dramatic upregulation at 18 hpi. This pattern suggests they were initially inhibited by the EPN, creating a critical window for infection establishment, before being hyperactivated in a final, concerted immune effort by the host. Conversely, genes in the lower section (Cluster 3), including *phenoloxidase* (a key melanization enzyme), the stress-responsive *dual oxidase*, the basement membrane component *laminin*, and the autophagy-related gene *ATG18a*, were persistently suppressed. This sustained downregulation likely benefits the EPN by impairing humoral melanization, tissue repair, and cellular clearance mechanisms.

The JNK cascade revealed a similar dichotomy. Cluster 2 genes, such as the stress sensor *GADD45α*, the transcription factor *Jra* (Jun-related antigen), and kinases *Btk* and *Shark*, were suppressed early but highly induced late. Their late upregulation may represent a desperate host attempt to activate pro-apoptotic or inflammatory JNK signaling to eliminate infected cells. In contrast, Cluster 3 genes, including the ubiquitin-conjugating enzyme *Ube2N*, the small GTPase *Rac1*, the signaling molecule *Wnt2*, the ubiquitin ligase adapter *Roadkill*, and the immune adapter *TRAF4*, were suppressed throughout infection. The continuous inhibition of these positive regulators likely cripples the host’s ability to mount an effective JNK-mediated immune response, thereby facilitating EPN success. The distinct regulation of specific components within this cascade highlights its pivotal, yet vulnerable, role in coordinating insect immunity.

Finally, the phagocytosis illustrated the conflict over cellular defense. In Cluster 2, genes such as the phagocytic receptor *Draper*, the transcription factor *Relish*, and the chloride channel *CIC-7* showed late-stage induction, potentially enhancing pathogen recognition and phagolysosomal clearance as a final line of cellular defense. On the other hand, Cluster 3 genes essential for phagosome maturation and cytoskeletal dynamics—*Rac1*, the lysosomal trafficking regulator *LYST*, and the extracellular matrix enzyme *Peroxidasin*—remained deeply suppressed. This sustained inhibition would severely compromise hemocyte-mediated engulfment and destruction of the nematode or its symbionts. The targeted suppression of these cellular machinery components underscores phagocytosis as a critical frontline defense that is strategically dismantled by the pathogen.

Collectively, these detailed expression profiles within shared immune pathways demonstrate that the infection outcome is determined not by the uniform regulation of entire pathways, but by the precise, antagonistic control of specific genetic components within the host’s defense network.

### 3.6. H. indica CQ7-2 Infection Orchestrates a Dynamic and Multifaceted Transcriptional Reprogramming of the T. absoluta Immune System

**Figure 6:**
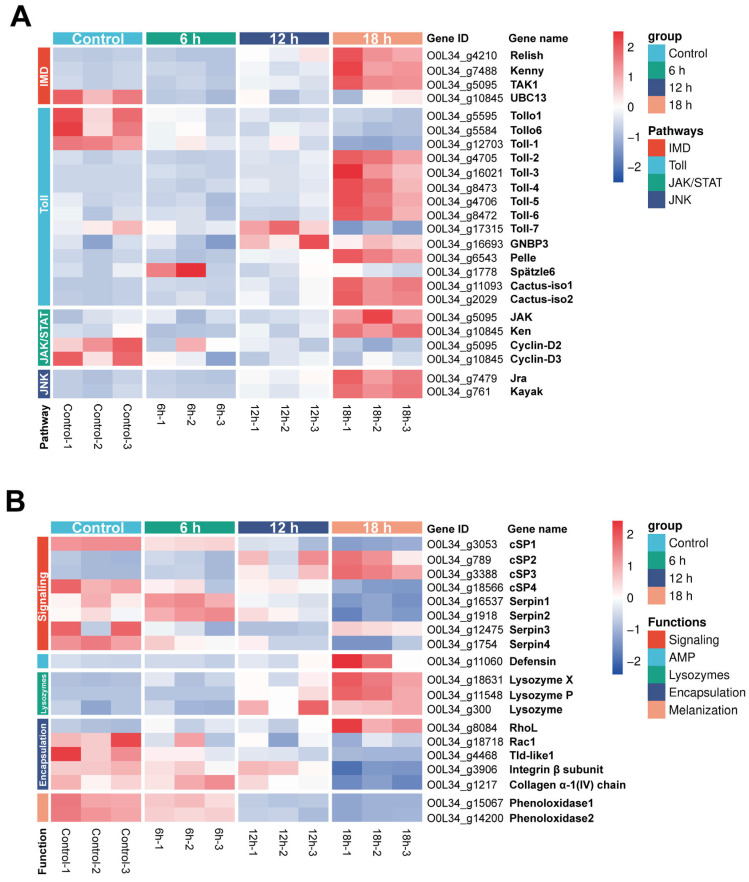
Temporal expression patterns of immune-related genes in *T. absoluta* larvae infected with *H. indica* strain CQ7-2. (**A**) Heatmap showing the expression profiles of key genes from major immune signaling pathways (IMD, Toll, JAK/STAT, and JNK) across infection time points (0, 6, 12, and 18 hpi). Genes are grouped by their pathway affiliation. Expression values are Z-score normalized across time for each gene, with red indicating upregulation and blue indicating downregulation (**B**) Heatmap displaying the expression dynamics of immune effector genes, categorized by their function in signaling (clip-domain serine proteases and serpins), antimicrobial peptides (AMPs), lysozymes, encapsulation, and melanization. Expression values are Z-score normalized; colors indicate relative changes within each gene.

To seek to delineate the immune strategies deployed by *T*. *absoluta* against *H*. *indica* CQ7-2 infection, we analyzed the expression patterns of key immune-related genes categorized by their involvement in specific pathways (Figure 6A) and core effector functions (Figure 6B). This approach revealed a complex temporal regulation of the host immune transcriptome, highlighting both the nematode’s suppression tactics and the host’s defensive countermeasures.

Analysis of major immune signaling pathways showed distinct transcriptional responses (Figure 6A). In the IMD pathway, central to antibacterial defense, key genes including the transcription factor *Relish* and upstream regulators *Kenny* and *TAK1* exhibited a classic Cluster 2 expression pattern, remaining stable until a sharp upregulation at 18 hpi. This suggests a potent, late-stage activation of the IMD pathway, likely as a last-ditch effort to combat the proliferating symbiotic bacteria of the nematode. In contrast, *UBC13*, a gene involved in NF-κB signaling within the pathway, was strongly suppressed from 6 hpi (Cluster 3), indicating a potential targeted disruption of a specific signaling node by the pathogen.

The Toll pathway, crucial for antifungal and anti-Gram-positive bacterial responses, displayed a more partitioned regulation. While the receptors *Tollo1* and *Tollo6*, along with *Toll-1*, were persistently downregulated (Cluster 3), the majority of downstream signaling components, including *Toll-2* to *Toll-6*, *Pelle*, and the adapter proteins *Castus-iso1* and *Castus-iso2*, were sharply induced at 18 hpi (Cluster 2). This dichotomy implies that the nematode or its symbiont may interfere with initial pathogen recognition at the membrane level, but the host retains the capacity to activate the intracellular signaling cascade at a late stage. The distinct expression of *Toll-7* (specific upregulation at 12 hpi) and the pattern recognition receptor *GNBP3* (sustained high expression from 12 hpi) points to the activation of a non-canonical Toll pathway branch, potentially in response to specific microbial patterns.

Within the JAK/STAT pathway, which regulates processes like epithelial immunity and wound healing, the key kinase *JAK* and the transcription factor *Ken* were upregulated late (Cluster 2). Conversely, *Cyclin-D2* and *Cyclin-D3*, genes linking STAT signaling to cell proliferation, were suppressed (Cluster 3). This pattern suggests a shift in JAK/STAT signaling away from tissue repair and towards immune effector production. Finally, in the JNK pathway, associated with stress responses and apoptosis, both the transcription factor *Jra* (a homolog of Jun) and *Kayak* (a homolog of Fos) were strongly induced at 18 hpi (Cluster 2), indicating a potent activation of stress and damage-response mechanisms during the late stages of infection.

Categorizing genes by their immune function further clarified the host-pathogen dynamics (Figure 6B). Among signaling molecules, clip-domain serine proteases (cSPs), which act as cascade components in prophenoloxidase (proPO) activation, showed divergent patterns: *cSP2* and *cSP3* were strongly upregulated late (Cluster 2), while *cSP1* and *cSP4* were progressively suppressed. Serine protease inhibitors (Serpins), which regulate these cascades, also showed varied responses, with *Serpin1/2* transiently induced early and *Serpin3/4* suppressed, suggesting a complex perturbation of the proteolytic networks that control immune activation.

Key antimicrobial peptides (AMPs) were represented by *Defensin*, which showed a strong late induction (Cluster 2), aligning with the concurrent activation of the IMD and Toll pathways. Similarly, all three lysozyme genes analyzed were sharply upregulated at 18 hpi (Cluster 2), indicating a robust but delayed attempt to mount a humoral antibacterial response.

Genes involved in cellular immune responses displayed targeted suppression. For encapsulation, a process critical for defending against large pathogens, the expression of cytoskeleton regulators was inhibited: *Rac1* and *Tld-like1* were suppressed from early time points, and *Integrin β subunit* and *Collagen α-1(IV) chain* plummeted at 18 hpi. Only *Rhol* followed the late-upregulation pattern. This widespread suppression likely severely compromises the host’s ability to form a cellular immune capsule around the nematode. Furthermore, the core enzymes of the melanization response, *Phenoloxidase1* and *Phenoloxidase2*, were progressively and strongly downregulated (Cluster 3). This targeted inhibition of phenoloxidase-activating cascades is consistent with a putative immune evasion mechanism in *H. indica* CQ7-2, which may impede the formation of toxic quinones and melanin detrimental to pathogens.

Collectively, the transcriptomic landscape revealed a multifaceted interaction. *H. indica* CQ7-2 was associated with transcriptional patterns consistent with immunosuppression, including pathogen recognition (specific Toll receptors), cellular encapsulation, and the melanization response. Conversely, the host mounted a vigorous, albeit potentially dysregulated, transcriptional counterattack at 18 hpi, activating systemic immune signaling (IMD, Toll, JAK/STAT pathways) and inducing humoral effectors like AMPs and lysozymes. However, this late-stage transcriptional upregulation of immune-related genes may be a correlate of host decline, rather than an effective defense. The data underscore that the outcome of infection was determined by the pathogen’s success in suppressing specific, critical components of the immune network, while the host attempts to compensate by hyper-activating other arms of its defense system.

## 4. Discussion

While the formidable threat of *T. absoluta* to global tomato production is well-established, and the potential of EPNs as biocontrol agents is recognized, the transcriptomic basis of the host-pathogen interaction—specifically *T. absoluta*’s defense responses to EPN infection and how EPNs modulate these defenses—remains largely unexplored. To date, comprehensive omics studies on *T. absoluta* have primarily focused on its interactions with host plants, transcriptomic responses to chemical pesticides, and chemical ecology, leaving a critical gap in understanding its molecular dialogue with pathogenic agents [43,44,45,46]. Our research addresses this gap by providing the first comprehensive temporal transcriptomic analysis of *T. absoluta* larvae during infection by the native Chinese EPN strain *H*. *indica* CQ7-2. It should be noted that pooling RNA from 10 larvae per sample may mask individual variability, though it was practical for transcriptomic profiling. We characterize the sequential transcriptional reprogramming of the host immune system and demonstrate that the low LC_50_ of *H. indica* CQ7-2 is correlated with modulation of key components in the immune network. Such transcriptional modulation of specific components in the host’s defense network culminates in a dysregulated transcriptional response of immune-related genes, which may potentially facilitate the pathogen’s successful establishment in the host.

The insect immune system is regulated by a complex array of signaling pathways, which orchestrate a diverse set of specialized defense mechanisms broadly categorized into humoral and cellular immunity. These mechanisms operate both locally and systemically to combat pathogen invasion [47]. In model insects such as *Drosophila melanogaster*, infection with EPNs like *H*. *bacteriophora* triggers a rapid immune response, characterized by the upregulation of key components of the IMD pathway (e.g., the transcription factor *Relish*), various antimicrobial peptides (AMPs), pattern recognition receptors, and other immune-induced molecules within hours post-infection [48]. However, the effectiveness of the host’s immune response depends critically on the EPN’s ability to evade or suppress immunity, as evidenced by the strong immune response observed in *Octodonta nipae* compared to the weak response in *Helicoverpa zea* [49,50]. The nematode’s role in actively suppressing host immunity is further supported by experiments involving axenic (symbiont-free) *H. bacteriophora*, which lead to the downregulation of AMP and lysozyme genes, whereas challenge with the symbiotic bacteria (*Photorhabdus*) alone provokes a strong upregulation of these genes [51]. This indicates that the nematode itself can initiate immunosuppression, a notion corroborated by research showing that secretions from *H. bacteriophora* during early infection can inhibit the expression of key immune effectors, suggesting an evolved strategy to create a window for bacterial proliferation [52,53].

In our study on *T*. *absoluta*, the interaction with *H. indica* CQ7-2 exhibited a distinct dynamic. Specifically, the majority of humoral immune genes associated with the IMD and Toll pathways remained unresponsive during the early and middle stages of infection (6 and 12 hpi). Notably, the expression of several key genes, including *UBC13*, *Tollo1*, *Tollo6*, and *Toll-1*, was even suppressed during this period. Although a substantial number of these immune genes were significantly upregulated by the late infection stage (18 hpi), this surge was insufficient to prevent host mortality, particularly as the expression of some critical genes remained suppressed (Figure 6A). This pattern demonstrates that a key aspect of *H. indica* CQ7-2′s pathogenicity is its ability to successfully evade the host’s humoral immune response during the critical early and mid-phase of infection through targeted suppression of key genes.

Insect cellular immunity, mediated by hemocytes, constitutes a critical defense line against large pathogens such as parasitic nematodes, fungi, and parasitoid wasps through phagocytosis, encapsulation, and melanization [54,55,56]. For instance, previous studies have demonstrated that *S*. *feltiae* can induce broad immunosuppression in *G*. *mellonella*, characterized by rapid inactivation of the prophenoloxidase system within 30 min, suppression of both encapsulation and phagocytosis within 2 h, and ultimate downregulation of antimicrobial genes via parasite-host interacting proteins (HIPs) [57]. Our transcriptomic data indicate that *H. indica* CQ7-2 is associated with dysregulation of the coordinated cellular immune responses in *T. absoluta*, corresponding to temporal downregulation of key components involved in these defense processes. The impairment begins at the initial stage of pathogen recognition: although the key recognition factors *PGRP-LB* and *GNBP3* were upregulated at 18 hpi and 12/18 hpi, respectively, this delayed activation failed to trigger an immediate immune response [58]. Instead, the comprehensive activation of major immune pathways was delayed until the host was near death, thereby creating a critical window for successful immune evasion by the nematode. Subsequently, the suppression extended to hemocyte proliferation and differentiation. Genes encoding central immune regulators—such as the transcription factor *Relish* (IMD pathway) and the signaling components *JAK* and *Ken* (JAK/STAT pathway)—along with effectors like *Cyclin-D2* and *Cyclin-D3*, remained unactivated until a sharp upregulation occurred shortly before host death (18 hpi; Figure 6A) [59]. This delayed activation likely represents a last-ditch, ineffective host effort rather than a successful defense, suggesting that the nematode’s early suppression is decisive.

Furthermore, the execution phase of encapsulation and melanization was profoundly compromised. The migration of hemocytes and cytoskeletal rearrangement, governed by small GTPases such as *RhoL* and *Rac1*, were impaired [60]. While *RhoL* was only induced late in the infection, *Rac1* expression was persistently suppressed. Crucially, genes encoding structural components necessary for forming the encapsulation capsule, such as *Integrin β subunit* and *Collagen α-1 (IV) chain*, along with *Tld-like1*, a gene involved in melanization activation, were consistently downregulated throughout the infection (Figure 6B) [61,62]. This sustained suppression likely creates a safe niche for the nematode and its symbiotic bacteria within the host hemocoel.

Further analysis of the Cluster 3 expression profile—characterized by sustained downregulation from early infection—provided strong evidence for a broad suppression of cellular immunity (Figure 5). Genes within this cluster that were enriched for the “defense response” GO term were predominantly associated with cellular immune functions. These included *Phenoloxidase1/2*, the key enzymes catalyzing the melanization cascade; *Dual oxidase2*, which produces reactive oxygen species (ROS) for oxidative killing; and effectors of cellular phagocytosis such as *ATG18a* (autophagy-related) [56,63,64]. Even the glucose-6-phosphate enzyme (*G6PE*), which may support the energetic demands of immune activation, was suppressed [65]. The coordinated downregulation of these critical components suggests a complex interaction between *H. indica* CQ7-2 and the host’s cellular immune system, where transcriptional suppression of key cellular immune factors may create favorable conditions potentially supporting the nematode’s establishment and proliferation in *T. absoluta*.

## 5. Conclusions

In conclusion, this study provides the first comprehensive temporal transcriptome atlas elucidating the molecular arms race between the invasive pest *T*. *absoluta* and the native EPN *H*. *indica* CQ7-2. We identify an association between CQ7-2 infection and multi-layered transcriptional changes in *T. absoluta*’s immune system, corresponding to sequential alterations in the host’s humoral and cellular defense pathways—including key components involved in pathogen recognition, hemocyte proliferation, and the melanization and encapsulation effector mechanisms. The transcriptomic evidence is consistent with targeted suppression of host immunity, providing a basis for mechanistic hypotheses that require functional validation. These findings not only advance our understanding of the molecular basis of EPN-host interactions in a non-model system—specifically by delineating the temporal transcriptional reprogramming of *T. absoluta*’s immune network and its modulation by EPNs—but also establish CQ7-2 as a promising, locally sourced biocontrol agent, given its low LC_50_ against target larvae and transcriptomic evidence of targeted interference with key host defense pathways. Moving forward, future work should focus on functional validation of key candidate genes identified here (e.g., via RNAi) and explore the potential for synergistic applications that enhance the efficacy of CQ7-2 in integrated pest management strategies against *T. absoluta*.

## Data Availability

The data presented in this study are available in article and Supplementary Material. The raw sequence data reported in this paper is openly available in NGDC GSA database (https://ngdc.cncb.ac.cn/gsa/search?searchTerm=CRA036293 (accessed on 2 January 2026)).

## Supplementary Materials

The following supporting information can be downloaded at: https://www.mdpi.com/article/10.3390/insects17030240/s1, Figure S1: Quantitative real-time PCR validation; Figure S2: Sample similarity heatmap of RNA-seq samples; Figure S3: Principal component analysis (PCA) of transcriptomic profiles across all samples; Table S1: Log-rank test results comparing survival distributions between biological replicates; Table S2: Primer sequences of quantitative real-time PCR; Table S3: Virulence of *Heterorhabditis indica* CQ7-2 against *Tuta absoluta*; Excel: Processed count matrices.
