# Supplementary material for: Biocontrol Mechanisms of a Chinese Heterorhabditis indica Strain Against Tuta absoluta: Virulence Assay and Time-Course Transcriptomics of Host Immune Responses
Source: Insects. 2026 Feb 26;17(3):240. doi: 10.3390/insects17030240 (PMC13027325; doi:10.3390/insects17030240)
Supplement: Supplementary file 1 [file insects-17-00240-s001.zip › Supplementary Material-revised.pdf]

Figure S1

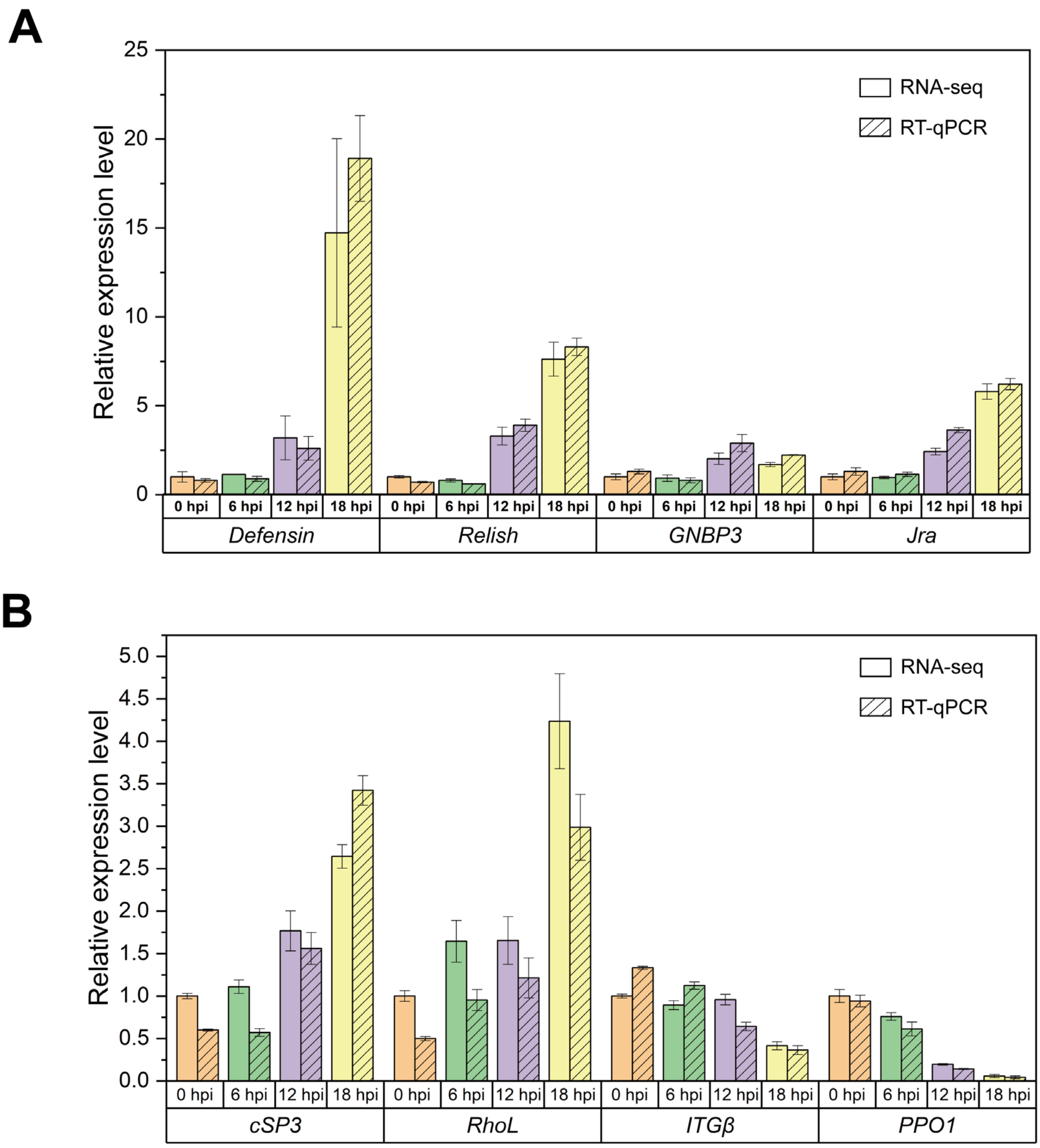

Figure S1. Quantitative real-time PCR validation. *Tuta absoluta* genes (O0L34_g11060, O0L34_g4210, O0L34_g16693, O0L34_g7479) **(A)** and genes (O0L34_g3388, O0L34_g8084, O0L34_g3906, O0L34_g15067) **(B)** were selected from the RNAseq dataset and qRT-PCR results. Open bars represent transcript levels obtained from RNAseq; bars with a diagonal pattern show expression levels obtained from qRT-PCR experiments.

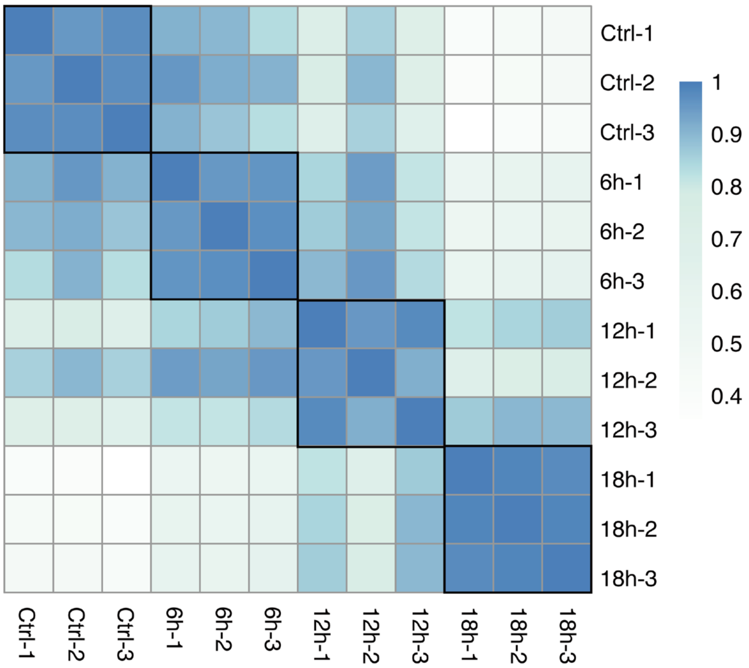

Figure S2. Sample similarity heatmap of RNA-seq samples. Pairwise sample distances were computed from normalized transcriptome data and visualized via a heatmap. Dark blue color denotes high sample similarity. All biological replicates within the same time point (Control, 6 h, 12 h, 18 h) cluster tightly together, confirming excellent reproducibility.

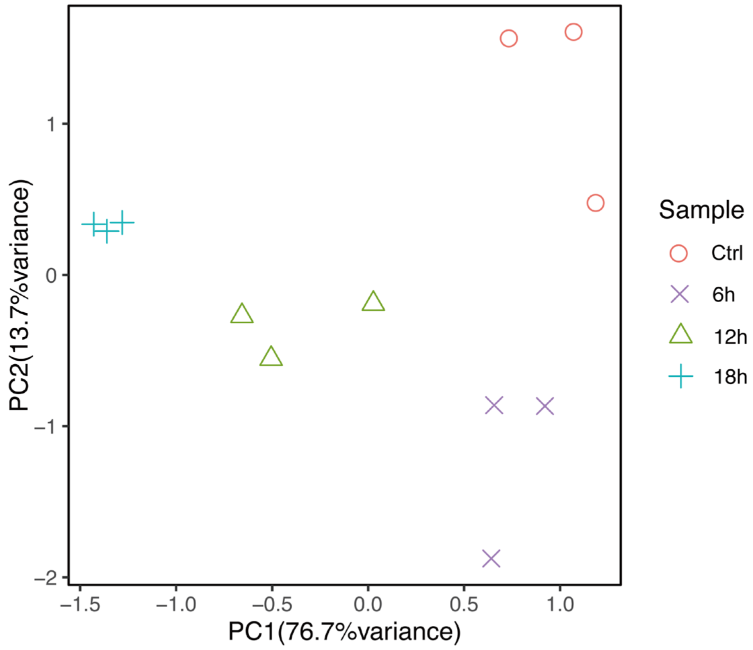

Figure S3. Principal component analysis (PCA) of transcriptomic profiles across all samples. PCA was performed based on the variance-stabilizing transformed (VST) read counts to reduce technical noise. Each point represents a biological replicate, colored and shaped according to the sample group (Control, 6 h, 12 h, and 18 h post-treatment).

Table S1. Log-rank test results comparing survival distributions between biological replicates

Test insect species	Test EPN species	Concentration (IJs/larva)	Log-rank (Mantel-Cox) test		
			DF	χ^2	*p*-value
T. absoluta	*H. indica* CQ7-2	0	2	0.263	0.8766
		5	2	0.840	0.6571
		10	2	0.633	0.7288
		20	2	0.523	0.7607
		40	2	1.232	0.5400
		80	2	/	/

Non-significant *p*-values ($p > 0.05$) confirm inter-replicate consistency.

Table S2. Primer sequences of quantitative real-time PCR

GeneBank ID	Gene name	Orientation	Primer sequences
O0L34_g11060	*Defensin*	F	AAGAGAGTCGTCAAGCTCGT
		R	TCCTTTGTACCCCTTCCATATGCAG
O0L34_g4210	*Relish*	F	ACCACTGCACAAAATAAAACTAGAACTCACC
		R	TGAGCCAGCCGTACCTTGATCACA
O0L34_g16693	*GNBP3*	F	ACGGAAATCTGGTCATCACGCCTA
		R	GCATTGAGTCGTATCTACTGCACCT
O0L34_g7479	*Jra*	F	TACCCGCAGCCCATAGTCAAGGACGA
		R	ACTCGATTTCTCTGCCGTTTGCGTTC
O0L34_g3388	*cSP3*	F	ACCCACGACAAATAACAACAGACCA
		R	CCACCCCATATTCTGTCGCTCTCC
O0L34_g8084	*RhoL*	F	TGAACTGAAACACTTTTGCGCCTCC
		R	CCGAGCACTCCACGAAGCC
O0L34_g3906	*Integrin β subunit*	F	AAATTGACTCTAACCCCTGACTGC
		R	ACCAGTAGGCTTTATAATGAACGACT
O0L34_g15067	*Phenoloxidase 1*	F	TGGAGTCAACCTGCACCACTACCAC
		R	GCATGTAGAAGAACAGCTCCCCACGAC
	EF1α	F	CCCATACAGTGAATCCCGTTTCG
		R	TTGTCTCCGTGCCATCCAGAA

Table S3. Virulence of *Heterorhabditis indica* CQ7-2 against *Tuta absoluta*

Test insect	Stage	LC_{50} (IJs/larva) (95% confidence interval)	LC_{90} (IJs/larva) (95% confidence interval)
Tuta absoluta	*4th instar*	1.35 (0.092 – 3.23)	19.07 (11.97 – 41.27)

* LC_{50}: the median lethal concentration 84 hours after the infection treatments (5, 10, 20, 40 and 80 IJs/larva). IJ: infective juvenile, the third-stage larval form of entomopathogenic nematodes, which is the only free-living stage.
